# Health System Considerations for Community-Based Implementation of Automated Respiratory Counters to Identify Childhood Pneumonia in 5 Regions of Ethiopia: A Qualitative Study

**DOI:** 10.34172/ijhpm.2023.7385

**Published:** 2023-10-18

**Authors:** Angeli Rawat, Agazi Ameha, Jonas Karlström, Lisanu Taddesse, Elias Legesse Negeri, Anne Detjen, Kristoffer Gandrup-Marino, Noah Mataruse, Karin Källander, Abraham Tariku

**Affiliations:** ^1^UNICEF Supply Division Innovation Unit, Copenhagen, Denmark.; ^2^School of Population and Public Health, University of British Columbia, Vancouver, BC, Canada.; ^3^UNICEF Ethiopia Country Office, Addis Ababa, Ethiopia.; ^4^Global Programmes and Research, SingHealth Duke-NUS Global Health Institute, Duke-NUS, Singapore, Singapore.; ^5^Federal Ministry of Health of Ethiopia, Addis Ababa, Ethiopia.; ^6^Frontieri Consult, Addis Ababa, Ethiopia.; ^7^Child and Community Health Unit, Health Programme Group, UNICEF, New York City, NY, USA.; ^8^UNICEF Supply Division, Copenhagen, Denmark.; ^9^Digital Health and Health Information Systems Unit, Health Programme Group, UNICEF, New York City, NY, USA.; ^10^Department of Global Public Health, Karolinska Institutet, Stockholm, Sweden.

**Keywords:** Childhood Pneumonia, Diagnostic Aids, Implementation Research, Ethiopia, Respiratory Rate Counting

## Abstract

**Background:** In Ethiopia, childhood pneumonia is diagnosed in primary healthcare settings by measuring respiratory rate (RR) along with the presence of cough, chest indrawing, difficulty breathing, and fast breathing. Our aim was to identify health system-level lessons from implementing two automated RR counters, Children’s Automated Respiration Monitor (ChARM) by Phillips^®^ and Rad-G by Masimo^®^, to provide considerations for integrating such devices into child health programmes and health systems. This study was part of an initiative called *the Acute Respiratory Infection Diagnostic Aids (ARIDA)*.

**Methods:** Key informant interviews (KIIs) were conducted with 57 participants (health workers in communities and facilities, trainers of health workers, district management, and key decision-makers) in five regions of Ethiopia. Data were analyzed in ATLAS.ti using thematic content analysis and themes were categorized using the Tanahashi bottleneck analysis.

**Results:** All participants recommended scaling up the ARIDA initiative nationally as part of Integrated Management of Newborn and Childhood Illness (IMNCI) in primary healthcare. Health workers perceived the devices as: time saving, acceptable by parents and children, and facilitating diagnosis and referrals. Health workers perceived an increased demand for services and reduced numbers of sick children not seeking care. Participants recommended increasing the number of devices distributed and health workers trained. Strengthening drug supply chains, improving oxygen gas availability, and strengthening referral networks would maximize perceived benefits. While training improved knowledge, more supportive supervision, integration with current guidelines and more guidance related to community engagement was recommended.

**Conclusion:** Automatic RR counters for the decentralized diagnosis of childhood pneumonia could have positive impact on improving the quality of diagnosis and management of pneumonia in children. However, the study has shown that a health system approach is required to ensure all steps along the pneumonia pathway are adequate, including drug and oxygen supply, community engagement, health worker training and support, and referral pathways.

## Background

**Key Messages**

**Implications for policy makers**

- Automatic respiratory rate (RR) counters for the decentralized diagnosis of childhood pneumonia could have a positive impact on improving the quality of diagnosis and management of pneumonia in children.
- However, the study has shown that a health system approach is required to ensure all steps along the pneumonia care pathway are adequate, including drug and oxygen supply, community engagement, health worker training and support, and referral pathways.
- The introduction and scaling of innovations (such as automated RR counters) provides an opportunity for broader health system strengthening of all relevant components of the pneumonia care continuum.

**Implications for the public**

Automatic respiratory rate (RR) counters for the decentralized diagnosis of childhood pneumonia have the potential to improve the quality of diagnosis and management of pneumonia in children. This is particularly important in the community settings and at primary care levels which are often the first points of contact of patients with their health systems. The study highlights the need for adequate training, drug and oxygen supply, community engagement, health worker training and support, and referral pathways. The introduction and scaling of innovations (such as automated RR counters) provides an opportunity for broader health system strengthening of all relevant components of the pneumonia care continuum. This, in turn could improve early diagnosis, management and treatment for children with pneumonia.

Childhood pneumonia, the leading infectious cause of death among children under 5 years of age globally, contributed to 740 000 deaths in children in 2019.^1^ In low- and middle-income countries (LMICs), childhood pneumonia diagnosis, classification and case management are guided by measuring respiratory rates (RRs) (ie, manually counting thoracic movements and breaths for 60 seconds) in children with cough and/or difficult breathing or chest indrawing. This approach identifies fast-breathing and determines whether children are treated with antibiotics, referred for specialized care and/or oxygen therapy.^2^ Manually counting RR has limited accuracy, high variability, and incorrectly measuring and recording RR can lead to misdiagnosis, inappropriate antibiotic use and delayed referrals.^3-7^ Innovations such as automated RR counters could potentially improve the quality of care for childhood pneumonia at the primary healthcare level.^8,9^

 At the time of the study, two automated RR counters were commercially available: Children’s Automated Respiration Monitor (ChARM) by Phillips^®^ (measures RR only) and Rad-G by Masimo^®^ (a multimodal device that measures RR and pulse oximetry).^9^ ChARM, which is no longer in production, has shown moderate RR count agreement when compared to RR measurements from a video panel of experts.^10^ It was feasible and easy to use within Integrated Management of Newborn and Childhood Illness (IMNCI) and integrated Community Case Management (iCCM) platforms, with field trials demonstrating that facility and community health workers correctly adhered to assessment steps.^11-13^ Strong agreement has been shown between automated RR measurements from Rad-G and paediatrician’s manual RR counting in India^14^ but lower agreement has been observed in the Democratic Republic of Congo, especially for fast breathing.^15^ Multimodal devices such as Rad-G, which include pulse oximetry in addition to RR measurement, could potentially improve rates of correct management of severe childhood pneumonia cases by 44% within IMNCI.^16^ The addition of temperature and oxygen saturation to RR measurement has also been shown to improve the specificity of diagnosing childhood pneumonia.^17^

 While innovations like automated RR counters address a key bottleneck related to childhood pneumonia diagnosis, other bottlenecks along the pneumonia care pathway still remain (eg, delayed care seeking, lack of access to quality healthcare with skilled providers and uninterrupted stock of essential commodities) and increase children’s risk of severe illness and death from pneumonia and other childhood illnesses.^18-20^

###  The Acute Respiratory Infection Diagnostic Aids Initiative in Ethiopia

 Between 2015-2019 a multipronged approach was launched in Ethiopia to increase oxygen in public hospitals via policy development, procurement and maintenance of oxygen equipment, and training healthcare workers. A study of 32 national hospitals indicated that during this time, the functional availability of pulse oximetry increased from 45%-86%.^21^ In addition, the Ethiopian government implemented the Acute Respiratory Infection Diagnostic Aids (ARIDA) initiative supported by United Nations Children’s Emergency Fund (UNICEF). The aim of the ARIDA initiative was to identify and test innovative diagnostic tools to improve the identification, classification and management of children with pneumonia. Under ARIDA, the use of two automated RR counters, the ChARM (RR only) and the RadG (RR and pulse oximetry) were evaluated within the context of iCCM/IMNCI at primary-level health facilities (eg, hospitals, health centres and health posts) and in communities in Ethiopia.^22^

 The ARIDA initiative was implemented in 5 regions of Ethiopia (from November 2018 to April 2019). Health extension workers (HEWs) who diagnose children with pneumonia at community-based health posts as well as at patient homes, and Front-line health workers (FLHWs) based in facilities were trained. Trainings and refresher trainings covered pneumonia diagnosis and management and the correct application of the devices. Trainings lasted one to one and a half days using the Training of Trainers model and included training guides along with job aids that were provided to each trainee. Training was given to 5291 health workers (3911 HEWs from 1973 health posts were trained on ChARM; 1380 FLHWs in over 445 health centres and primary hospitals were trained on Rad-G).

 This initiative resulted in an estimated 279 750 direct child beneficiaries. Detailed methods of the ARIDA initiative have been described previously.^9^ iCCM and IMNCI guidelines were updated to include use of the ARIDA devices.^23^ Regional health bureaus allocated devices. District Health Office supervisors who completed the training provided monthly supportive supervision to the districts and the facilities. UNICEF field and central offices performed at least two site visits during the project. The first visit occurred within a month of the training and receipt of the devices and the second occurred during the middle of the project implementation.

 The results of the usability and acceptability of the ARIDA initiative have been published elsewhere.^12,13,24,25^

 This study aimed to identify practical health system-level lessons from the implementation of the ARIDA initiative. Objectives included understanding successes, challenges and recommendations for implementation from multiple perspectives within the health system and to identify further evidence needed for scale-up.

## Methods

 This study’s approach builds upon the World Health Organization (WHO) health system building blocks and the conceptual implementation research framework by Peters et al.^26,27^ We applied a modified Tanahashi Bottleneck Framework for the evaluation.^28^ We utilized semi-structured key informant interviews (KIIs) to meet the research objectives. KIIs were conducted using interview guides tailored to participants’ expertise and informed by current literature in consultation with the UNICEF Ethiopia country office and the Federal Ministry of Health of Ethiopia (FMoH). The guides included themes related to use of devices for RR counting, health workforce perceptions, training and supportive supervision, referral pathways, availability of supplies and commodities for pneumonia (eg, amoxicillin, gentamicin and oxygen), community uptake of the ARIDA initiative (including community engagement and perceived caregivers’ responses) and scaling up within the health system. The guides were not pilot tested. Recruitment, data collection and data quality monitoring were completed by trained researchers in coordination with UNICEF. Members of the research team were male, with Masters degrees or undergraduate degrees, trained in data collection, qualitative research methods, and the objectives of the study. Interviewer requirements included being male or female, having university level education related to health sciences, had no prior relationship with or knowledge about the interviewees and no reported biases. Participants had no prior knowledge of the researcher, other than knowing the objectives of the research. No interviewer biases were identified.

 Criterion purposive sampling was used to identify participants in each region from HEWs in communities, facilities (eg, FLHWs, clinic managers), trainers of the health workers, and district management/decision-makers (eg, *woreda*- and zonal-level management, regional key decision-makers and policy influencers) until a pre-determined number was reached. Participants were approached face-to-face, provided with information (verbally and in writing) on the objectives of the research and the voluntary nature of participation. All participants provided written, informed consent, and their identities were kept confidential. Interviews lasted between 15 to 78 minutes. Interviews were conducted at the interviewees’ offices in their preferred language (Amharic, Tigrigna or Afan Oromo), with no other people present. KIIs were audio recorded and field notes were taken. Audio files were transcribed and translated into English. Data quality and translation consistency were checked across the study process by the research team. Due to time limitations, the transcripts were not returned to participants for confirmation. No repeat interviews were done and no one refused to participate. The aim of the study was to reach saturation within participant groups across diverse perspectives in the health system and geographic areas within the timeframe and budget of the study.

 The study was designed and coordinated in partnership with the FMoH and adhered to ethical standards of embedded implementation research.^29^ Institutional review board approval was not sought as the study was part of routine program evaluation. All regional health bureaus provided letters of support endorsing the qualitative data collection. FMoH partners and regional bureau heads reviewed and approved the consent forms and study. Written, informed consent was collected from all participants and no identifying, personal information was reported. All data were kept secure in password protected files.

###  Participants

 Participants (n = 57) were interviewed between June 10, 2019 and July 4, 2019 across five regions, seven zones and 14 *woredas* where the ARIDA initiative was implemented. The regions included Benishangul-Gumuz, Oromia, Southern Nations, Nationalities, and Peoples’ Region (SNNPR), Tigray, and Afar. The regions with the largest numbers of participants were SNNPR (30%, n = 17) and Oromia (26%, n = 15), coinciding with the areas where the largest scale of activities occurred (Table 1). Participants were interviewed an average of 4 months after ARIDA implementation (range: 3-5 months).

**Table 1:** Participants by Geography

**Region**	**Zone**	**Woredas**	**n**	**Months Since Implementation at the** **Time of the Interview**
Afar	Zones 1 and 4	Dubti and Asayita	8	5
Benishangul-Gumuz	Asossa	Asossa and Bambasi	8	4
Southern Nations, Nationalities, and Peoples’ Region	Kaffa	Chena and Gnimbo	7	4
	Sidama	Bona Zuria and Bensa	10	4
Tigray	Central	Adwa and Laelay Maichew	9	4
Oromia	East Hararghe	Gursum and Jarso	8	3
	West Shoa	Ambo Zuria and Ada’a Berga	7	3
		**Total**	57	

 The majority of participants were health workers, including HEWs (n = 14) and FLHWs (n = 14), health centre managers (n = 7) and trainers of the health workers (n = 7). Participants also included Zonal or regional health system managers (n = 5) and policy influencers and decision-makers (n = 10), which included senior management from the FMoH (Table 2). The HEWs interviewed covered an average of 1600 households (range: 144 to 7754).

**Table 2:** Participants by Role in ARIDA Implementation

**Role**	**n**
HEW	14
Facility level health worker	14
Health centre manager	7
Regional and zonal health system manager	5
Trainers of health workers	7
Policy influencer and decision-maker	10
**Total**	**57**

Abbreviations: ARIDA, Acute Respiratory Infection Diagnostic Aids; HEW, health extension worker.

###  Data Handling and Analysis

 English transcripts were analyzed by a trained researcher (AR) utilizing ATLAS.ti version 8 software (Scientific Software Development GmbH). Open, first-level coding was done based on the *a priori* sub-categories of the KII guides using a deductive approach. No new emergent themes were identified. This resulted in 1271 unique open codes which were applied to a modified Tanahashi bottleneck analysis framework,^28^ resulting in 58 codes. The Tanahashi framework focuses on the interactions between service provision and the end service users. We modified the framework with an emphasis on ascertaining health system successes and challenges related to the implementation of the ARIDA initiative, within the following domains: (1) Supply; (2) Demand; (3) Enabling environment; and (4) Quality (see Supplementary file 1).

 Co-authors (JK and AA) confirmed the correct application of the framework. For codes in the ‘supply’ category, data were disaggregated by pastoralist/semi-pastoralist regions (eg, Afar and Benishangul-Gumuz) and non-pastoralist regions in order to identify geographical differences in the supply chains or access to commodities across these parameters. Additionally, health-seeking behaviour was disaggregated by participant category. Differences that were identified in these sub-analyses are described in the results section. Data were synthesized by participant categories, and themes related to the specific objectives of the research were identified utilizing the grounded theory approach.^30^

## Results

###  Devices-ChARM (Supply and Quality)

 The majority of participants perceived the ChARM device to be more accurate than manual counting using timers for counting RRs to diagnose pneumonia in children. Participants also described ChARM as easy to use and read, with a clear indication of when to refer the patient for further care, as seen in the following quote:

 “*Before ChARM, we used to count children’s breathing using our eyes. But now ChARM made breath counting easier. It is faster than the previous method because it can count the child’s breaths immediately. The previous method could lead us to make a wrong decision. ChARM displays the correct count [and] is more accurate because [it] is supported with a diagnostic tool. It displays the correct figure/count”* [HEW, SNNPR].

 Health workers spoke of benefits related to having age-specific or standardized breathing counts shown in the device, which included improving their confidence to diagnose and manage pneumonia while reducing unnecessary referrals and prescriptions. Some health workers felt the ChARM device saved time or was faster compared to previously used timers and described advantages of completing other tasks while awaiting the results (eg, intake forms, other vitals, charting). HEWs described ChARM as lightweight and easy to transport during household visits.

 However, inadequate numbers of ChARM devices was identified as a challenge since every health worker did not receive a device, especially in pastoralist regions, as seen in the following quote:

 “*We do not have enough devices for the number of health extension workers. We are facing problems when we visit households with sick children”* [HEW, SNNPR].

 Participants recommended that all health workers be provided with a device. Health workers also described increased consultation times using ChARM when the child was moving or agitated, severely malnourished, or fearful of the device, especially in pastoralist areas. They recommended a device that would function in these circumstances. Concerns were also raised about the battery life of the device, with mixed recommendations on preferences for chargeable or non-chargeable devices. Some felt the rechargeable devices allowed for continued use; others felt the non-rechargeable devices were better, as they were not influenced by the electricity fluctuations in the health centre.

###  Devices-RadRad-G (Supply and Quality)

 The majority of health workers felt that Rad-G was more accurate and faster than visually counting RR, with some describing it as a one-step device. Health workers felt it improved their knowledge to manage and treat pneumonia by providing more information (eg, age classification, classifying the severity of pneumonia, oxygen saturation, positive and negative results), and allowed them to diagnose and refer based on evidence, as described below:

 “*In the case of the older method [visually counting], we treated with the help of signs according to a chart. Ages are [classified] as a total without [accounting for] age group characterization of cases. But the new device shows the readings according to age groups, which makes it preferable to use. The device gives a signal/message/if the case needs referral – a red light. The device itself tells you to refer the case and also shows whether cases are manageable at health centre level”* [FLHW, SNNPR].

 Challenges in using Rad-G included inadequate numbers of devices for the volume of patients since devices were concurrently used by multiple health facility departments (eg, outpatient and antenatal), as described below:

 “*Sufficient devices were not available in our health centre. We have one Rad-G which is not sufficient to serve the patients on time because of the high load of patients. If we get one additional device, two health workers can serve patients on time”* [FLHW, Oromia].

 Participants recommended providing additional devices and expanding training to all health workers and facilities, including health posts. Health workers recommended improving the functionality and sensitivity of Rad-G so the probe attachment could be easily used while children are moving or agitated. Some health workers felt Rad-G could be improved by reading temperature and pulse as well [Authors comment: Rad-G does measure pulse rate, but health workers were not trained on this as part of the ARIDA initiative]. Additionally, health workers were concerned that no device maintenance was scheduled and described issues related to device malfunction and charging (eg, lack of electricity, needing to monitor and charge the battery, device not being usable while charging). They recommended providing back-up batteries or increasing the battery life.

###  Caregivers’ and Children’s Reactions to the ARIDA Devices (Demand)

 Health workers reported that children had mixed reactions to the devices, identifying trends by age and geography, as described below:

 “*When children get diagnosed, they perceive the device as a toy. Even if children were crying before diagnosis, they get calm and relax when they see the device. All children do not react the same way while being diagnosed. We classify their ages into three groups. Children under age 1 react neutrally; children around age 3 usually perceive them as toys. There are also children who cry or react distressfully to the device”* [HEW, SNNPR].

 Participants felt 2–5-year-olds were more likely to be calmed or entertained by the devices compared to younger children, who may fear an unfamiliar provider or the possibility of injections. They described urban children as more likely to be entertained by images and sounds on the Rad-G devices, or view the devices as toys, compared to rural children, who presumably have less exposure to technology. Positive descriptions of reactions to the devices included words such as ‘calm, happy, or entertained,’ while negative reactions included ‘stressed/distressed, fear/frightened, angry/aggressive, or crying/fussing.’

 Despite some perceived negative reactions that health workers reported in their interactions with children, many health workers felt it had little impact on device use, aside from increasing consultation times. When children exhibited negative reactions health workers allowed caregivers to calm children (eg, breastfeeding), modified the device use (eg, hiding the device from the child’s view, turning off sound, and played with devices) or demonstrated its safe use on another child or adult.

 The majority of health workers felt caregivers reacted positively to the devices and often requested they use the devices since becoming aware of them from HEWs or through community engagement. Many health workers felt caregivers perceived devices to diagnose pneumonia more accurately, which increased parents’ trust in health workers’ diagnoses and capabilities, as seen in the quotes below:

 “*I contacted parents after treatment and asked them what they were told about the device. And they replied to me that formerly their children were clinically assessed by unconfirmed diagnosis, but after the device was introduced, that they are happy when they see the respiration is being counted correctly for their children”* [Regional Health Bureau representative, Benishangul-Gumuz].

 “*They feel very happy with our treatment of their children using the device. They say: ‘Did you have such a device before at health post level?’ The caregivers feel happy with it”* [HEW, Oromia].

 “*The caregivers react happily when I measure their children with the device. They feel that their children are being diagnosed correctly if they are measured by the device. They say: ‘My child got the right diagnosis with a right device and right treatment’”* [FLHW, Tigray].

 If caregivers had negative responses, health workers educated the caregivers and their communities on the benefits and demonstrated its safe use on children. Health workers felt the devices facilitated community diagnosis, saving travel time to facilities for rural patients.

###  Community Engagement (Supply)

 The aim of community engagement was to raise awareness about childhood pneumonia and introduce the availability of automated RR devices. Each region led their own engagement. It usually included HEWs informing communities via community networks (eg, health development armies, mothers’ groups, vaccination sessions, and health committees), during household visits, monthly meetings or at the health posts. Some community engagement was provided by nurses, in collaboration with the *woredas* and *kebeles*, or with written letters to the lower levels of the health system and communities (ie, village heads and administration, women’s associations). Another method was to rely on the word of mouth of caregivers visiting the health facilities. Lastly, some participants reported that there was no community engagement outreach done and that the community members were informed of the initiative when they visited the facilities.

 Many health workers reported high community acceptability of the engagement strategies, as demonstrated by perceived high attendance and seeking care when symptoms were present, as described below:

 “*Since [ARIDA] was implemented in our health post, more people who have sick children under 5 visit the health post for diagnosis and treatment. The community’s awareness about the device has increased. Whenever we go for outreach and whenever they come to the health facility, we teach them about the device, which created awareness*” [HEW, Oromia].

 “*When the community is aware of the device, it allows increased service utilization, which in our case resulted in increased health facility visits*” [Facility Manager, Benishangul-Gumuz].

 Some participants felt engaging communities (including HEWs) early and allowing for their recommendations on who should be engaged and how led to community ownership. This was described as improving trust with the communities, especially increasing the acceptance of HEWs as health workers.

 Many participants reported challenges related to disparities in community engagement activities, with some communities not receiving any engagement and unaware of the initiative or that free services were available at the facilities. They recommended utilizing community organizations and government structures to improve engagement and generate more demand for services. Some challenges to community engagement included low community participation for populations living in difficult-to-access geographical areas (eg, large distances to health facilities, mobile populations, and those impacted by floods), farmers during the harvest season and new mothers, as described below:

 “*Mothers who give birth for the first time may not be aware of this intervention, because these mothers have not participated in immunization sessions. We give health education on danger signs of pregnancy for pregnant mothers. Since we cannot address all mothers, we are unable to address all households”* [HEW, Tigray].

 Participants felt engaging schools, holding conferences targeting pregnant mothers and training community members (eg, HEWs and health development armies) could improve health education and community engagement, as described in the following quote:

 “*Yes, it is better to train health development armies and send them down to the community to train people under them. They use every opportunity to give health education while people are gathered together”* [HEW, SNNPR].

 Some participants described challenges related to lack of budgets for community engagement, including insufficient educational materials and incentives for participation. Participants recommended providing budget, strategic direction and planning for community engagement.

###  Initial Utilization/Health-Seeking Behavior (Demand)

 The majority of health workers perceived increases in care seeking behaviors at the community and the health facility levels, with some describing no changes. Many health centre managers and FLHWs reported increased patients seeking pneumonia-related care at the health centres since the initiative, as seen below.

 “*After Rad-G came to our facility, we noticed that even if the under-5 children get the common cold, they bring [them] immediately to our health centre. Recently, our community [has become] aware about the device – whenever they come to health centre they say: ‘Please see my child with this device’”* [FLHW, Oromia].

 Participants ascribed the increases to successful community mobilization and awareness, an interest in new technology, and community members sharing positive experiences after accessing health services with ARIDA devices. Many HEWs described how community members sought care for coughs earlier than before the initiative and felt being able to diagnose and treat children during home visits increased healthcare access to populations who would otherwise not attend the facilities. Many participants felt community members had more confidence or trust in diagnoses where devices were used and perceived increased quality of care received, especially for HEWs, as seen in the following quote:

 “*Before the introduction of the new equipment, patients did not believe in health extension workers’ treatment. But after the training, in collaboration with the woreda, different mobilization efforts were made to advertise and familiarize the device with the community. The number of pneumonia patients seeking treatment has relatively increased, both at facility and health extension level. Therefore, this innovation played a significant role in building trust in health workers from the community”* [Health Centre Manager, SNNPR].

 The challenges to seeking diagnosis and treatment for pneumonia and other childhood illnesses described by participants were related to access to health facilities and posts and a lack of drugs at the facilities. Many participants felt remote communities were unable to access services due to large distances to the facilities and health posts, poor road conditions, poor health post conditions and weak transportation networks. Some participants felt there were gaps in health workers’ knowledge of pneumonia treatment, which decreased the communities’ confidence in the health workers and reduced their motivation to engage in the initiative.

###  Drug Supply and Medical Oxygen (Supply)

 All participants confirmed that amoxicillin was available at the facilities but identified gaps with continuous and appropriate supply (ie, correct dosage and formulation). Many participants reported that gentamicin was not available in all health centers, especially in pastoralist areas. Stockouts of gentamicin were reported as lasting one month to over a year and were due to failure to order on time, lack of standardized supply chains, lack of budget and difficulties with drug importation, as described below:

 “*From the facilities, there is drug request [every] quarter or three months. We collect our share [of medication] and if there is lack of adequate drug, we buy [it] by allotting the budget of the health facility. Gentamicin is difficult to find. There are budget-related problems [i.e. lack of budget]. There is amoxicillin named DT tablet [that is] donated”* [Policy Influencer and Decision-Maker, Oromia].

 The most widely discussed impacts from drug stockouts were that patients were referred to higher-level facilities to collect drugs, required to purchase drugs, or children not receiving timely and appropriate treatment. Recommendations to improve the drug supply chain and prevent stockouts included stockpiling extra medication at health centres, early reporting and requesting of drug supplies, and timely availability of budget to purchase drugs. Many participants recommended strengthening the procurement and supply chains, and some recommended that the supply chain should be managed by the central government, rather than donors, to ensure harmonized coordination. Stockouts were described as compromising communities’ confidence and trust in health workers and the health system and decreasing health workers’ motivation.

 The majority of participants described a lack of oxygen at facilities where Rad-G devices were implemented or at linked referral facilities, despite the availability of cylinders for refill. Determinants of the lack of oxygen supply and its use included shortages of staff trained in oxygen administration and maintenance, lack of budget at health centres and within districts, and a national deficiency of supplies and equipment related to oxygen. Concerning the national distribution of oxygen, high-level participants discussed challenges related to poor transportation networks to provide oxygen to areas outside the central distribution points, shortages of cylinders and compressors, and poor communication between the government and stakeholders (eg, FMoH, implementing partners, etc), as described below:

 “*Coming from far to refill oxygen cylinders is very tedious and difficult. Even travelling 100 km to fill oxygen is difficult. So, to solve this problem the supply of the oxygen should be facilitated at the nearest centre. The capacity of the professionals should be upgraded – they must be equipped with the knowledge: how they administer, how they distribute it at the level of health station. They need to upscale their understanding. But the provision is the greatest problem”* [Policy Influencer and Decision-Maker, Oromia].

 “*In health centres, only an empty cylinder is available, with no oxygen … There are times when the children die because of a lack of oxygen. If a child has caught pneumonia, they need oxygen to breathe. The hospital may lack oxygen. But if oxygen is available at the health centre, there will be a chance to save the life of the child”* [Health Centre Manager, Oromia].

 The uninterrupted provision of oxygen was a widely discussed health system requirement for improving pneumonia case management. Many of the challenges identified are addressed in Ethiopia’s National Medical Oxygen and Pulse Oximetry Scale-Up roadmap (2016-2020/2021).^31^

###  Referral Pathways (Demand)

 Many participants felt referral pathways for children with severe pneumonia or hypoxemia functioned adequately after being diagnosed using ARIDA devices, despite describing many challenges. There were large variations in reported time and distance patients travelled when referred for oxygen therapy/treatment of severe pneumonia (range 3-75 km, average 17 km). The time it took to travel these distances ranged from 10 minutes (walk) to 1.5 hours (car), up to 3 hours (walk).

 Participants described successes with referral pathways including HEWs treating patients prior to referral and perceived reductions in unnecessary referrals to higher-level health facilities based on the devices’ readings. Many FLHWs felt that with the additional measure of pulse oximetry in RadG decisions to refer patients were easier than before. They felt health workers followed up with receiving hospitals appropriately.

 Challenges to the referral pathways were related to staff knowledge and motivation, transportation access, communication, inadequate referral forms and patient-related factors. Some participants felt health workers lacked knowledge of when to refer patients and motivation to refer patients, as it increased their already high workloads. Participants recommended that training on adequately filling out referral forms could facilitate referrals. Many participants described large distances, poor road conditions, and insufficient numbers and distributions of clinics as challenges to referrals. Providing functioning ambulances with fuel and improving road conditions were discussed as ways to facilitate timely and appropriate referrals. Communication was a determinant in strong referral networks and participants described gaps in communication between health facilities and hospitals to arrange referrals, especially concerning hospital bed availability. Health workers discussed a lack of stationery or forms for referrals, or forms that did not contain the necessary information, as challenges to referring patients. Some participants described challenges of patients not wanting to be referred, or patients bypassing the existing protocols and self-referring to higher-level facilities. This was often described as being related to the low knowledge levels of staff and the low quality of services provided at lower-level health facilities. Participants described how improvements in the supply of oxygen in facilities with Rad-G would reduce referrals. Participants recommended strengthening referral networks for timely referral of sick children. Recommendations included building the capacity of health workers, improving transportation and communication networks, educating the communities, ensuring adequate supplies and commodities, and more commitment from higher levels of the health system.

###  Staff Satisfaction and Motivation, Training and Supportive Supervision (Enabling Environment)

####  Staff Satisfaction and Motivation

 Health workers described being more satisfied and motivated since using the ARIDA devices and receiving the training, as seen below:

 “*It has a big influence. It has reduced my burden of work. Before, it was very difficult to count their beat [RR] because children could be unstable. But now, once we tie the instrument over the breast line, there is nothing to worry about. You just stop it when you see a red or green light. This has solved all these problems and enabled us to get an accurate count”* [HEW, Benishangul-Gumuz].

 “*This ARIDA package influenced our motivation and job satisfaction in positive ways. Since with full confidence, i.e., without any uncertainty we obtain accurate diagnostic results and save our time, we are happy with the device and more satisfied in our jobs”* [FLHW, Oromia].

 They attributed this to having increased confidence in their ability to diagnose and treat pneumonia and perceived simplification of their jobs. Many felt the devices saved time and allowed them to complete their jobs more efficiently while decreasing workload. HEWs felt it added value to their profession.

 Challenges related to staff satisfaction and motivation included increased workload due to more cases being screened and treated for pneumonia. Health workers also felt frustrated when they could diagnose but not treat patients due to a lack of treatments and health infrastructure. A few participants felt that devices malfunctioning or not being sufficiently charged for use compromised gains made by the initiative.

####  Training

 Health workers reported the training they received as part of the ARIDA initiative as highly acceptable and described it as practical, simple, accessible and participatory. The language used was clear, with adequate access to local languages and at appropriate levels for the health workers. The content was described as adequate to implement the devices and contained refreshers on MNCH (Maternal, Newborn, and Child Health) and IMNCI/iCCM. Health workers described facilitators to the training, which included the provision of chart booklets, practicing on volunteer children, and good mentorship and support throughout the training. Trainers were often described as well-qualified and accessible and some health workers described additional support from managers at facility, zonal and *woreda* levels.

 However, health workers felt more training days (eg, 3 days) were needed to be fully competent in correctly applying either of the two devices and making decisions on treatment and/or referral based on results. Some health workers felt the training needed more practical sessions (eg, demonstrating the use of devices in facilities and communities) and trainings. Many health workers recommended that more staff needed to be trained, ranging from HEWs on the device use at household levels to higher-level staff within the health system (ie, all facility, zonal and *kebele* managers). High staff turnover, low numbers of human resources and absenteeism were described as challenges to having only one health worker trained per facility.

####  Supportive Supervision

 Supportive supervision was described as having many benefits, including refreshing information learned, improving clinical practice, correcting mistakes and problem solving, identifying device malfunctions, and motivating staff. Many health workers reported receiving little or no supportive supervision; this was described as inadequate. Challenges to providing supportive supervision included lack of designated budget for supervision and no dedicated staff to provide technical support, not being requested to provide updates on the project successes or challenges, and a lack of management’s technical knowledge of the ARIDA initiative.

 Recommendations to improve supportive supervision included mandating supervision to occur monthly or every three months and including higher-level management in the process. Recommendations included federal-level support, providing budget and transportation to facilitate supervision, especially in rural areas, and improving reporting of the project throughout the health system.

###  Considerations for Scale-up (Demand)

 All participants recommended scaling-up nationally and integrating the devices into iCCM/IMNCI in all regions interviewed. The most widely discussed reasons for scale-up were that the intervention is perceived to save lives by improving early diagnosis and treatment for pneumonia and reducing pneumonia-related morbidity and mortality. Participants felt scale-up should be government-led with support from implementing partners (eg, inputs and logistics, including the purchase of devices). A few participants described the need for memorandums of understanding on the roles of stakeholders involved in scaling up ARIDA devices, as well as guidelines for use of the devices. Many participants also felt the intervention needed to be integrated and absorbed by the national health system in order to be sustainable and equitable.

 Many high-level participants involved with policy-making in the ministry felt the intervention could be easily integrated with IMNCI and iCCM guidelines given that the devices’ algorithms are congruent with diagnosis and treatment guidelines and with Ethiopia’s strong primary healthcare-based health system and efforts to improve quality of care. The most widely discussed challenges to integration were related to data collection and reporting (eg, lacking space in registers to record oxygen saturation, lack of data fields in the health information systems). Many higher-level participants felt that adequate financial resources existed to absorb the scale-up of ARIDA devices into the health system nationally, while fewer felt the budget for devices and training may hinder successes. Many participants described the need for adequate budget related to supplies (drugs, oxygen and device procurement) and human resources (training and follow-up).

 While all participants interviewed recommended scaling up nationally, further information is needed on costing with considerations for sustainability and scalability.

###  Policy Engagement and Evidence Needed (Enabling Environment)

 Ministry participants and policy-makers recommended engaging the following groups in policy dialogue: international implementing partners (including WHO, UNICEF, World Vision), non-governmental organizations, political leaders, FMoH, and researchers from universities. Input from lower levels of the health system was discussed as essential — including HEWs, health centre board members, health workers, pharmacists, primary healthcare unit staff and *kebele*, zonal and regional health bureaus. Engagement was recommended via technical working groups with roundtable discussions and experience sharing/workshops across all levels of the health systems.

 Participants identified the need for further evidence prior to health system scale-up and integration. This included evidence on the effectiveness of the devices in reducing mortality and morbidity. Participants recommended that evidence on acceptability and adaptability results from the pilot study be shared, including numbers of children identified and treated for pneumonia, clinics access to power, benefits of the devices and how they are being used, and health-seeking data. Participants from the Ministry identified the need for evidence, including return-on-investment studies, costing and forecasting that are adaptable by region, and how many people have been and need to be trained to render the services to the population.

## Discussion

 Practical health system-level lessons were identified from the implementation of the ARIDA initiative in 5 regions of Ethiopia. The newly-introduced automatic RR devices were acceptable by health workers and parents, and all participants recommended scale-up of the devices nationally. However, for the devices to have maximum impact on diagnosis and management of childhood pneumonia, considerations of scale-up must be accompanied by broader health system strengthening of all relevant components along the pneumonia and broader child healthcare pathway.

 Our study builds upon evidence from Ethiopia and Malawi, which has shown that the addition of pulse oximeters within Integrated Management of Childhood Illness can improve the identification of severe pneumonia and hypoxia in children.^32,33^ Furthermore, the results of our study suggest that the additional evidence provided by the devices could help to identify children with pneumonia who may not otherwise have visited healthcare facilities. Moreover, the provision of additional devices could improve equity and access to care while also increasing healthcare workers’ confidence in their ability to diagnose, treat, and refer pneumonia cases. While all of these factors suggest that the ARIDA devices have the potential to reduce pneumonia-related morbidity and mortality, several important considerations remain regarding widespread scale-up of this initiative.

 First, disparities in community engagement during the ARIDA initiative were identified. Effective community engagement and participation can lead to improvements in health-seeking behaviour in low resource settings.^34^ Engaging communities can ensure demand generation, improved care seeking and improving education during the implementation of automated RR counters. Equitable engagement should be facilitated through strategic community engagement guidelines and adequate budget for child health that includes leveraging and training existing community networks and groups to provide education and disseminate pneumonia related counselling.

 Second, although health workers felt the training and device implementation improved their job satisfaction and morale, health worker capacity must be considered. Our study findings are similar to a study in Ethiopia where the authors identified high acceptability and usability from health workers of Rad-G, but also highlighted the need for additional training.^24^ Training additional health workers in facilities and communities on the use of ARIDA devices would allow more health workers to use the devices simultaneously and create a more equitable workload distribution. However, supportive supervision and mentorship are critical determinants of the provision of consistent and high-quality child healthcare at decentralized levels of the health system,^35^ and these supports were not always adequate in our study. Dedicated resources for supportive supervision would facilitate uptake of the devices, but these resources may not be readily available in many regions.

 Third, additional resources must be allocated for pneumonia treatment and management in local health centres. Although amoxicillin was readily available during the study period, streamlining and strengthening supply chains for essential medicines in primary healthcare is recommended. In particular, there is an urgent need to improve access to medical oxygen in health centres by availing oxygen-related budget, providing training and supplies at all levels of the health system, supporting capacity building of human resources and developing decentralized oxygen distribution networks as described in the Oxygen Roadmap for Ethiopia.^31^ The availability of oxygen is especially important for care of children with severe pneumonia as well as other conditions, before, during referral and at referral level. As noted by participants in our study, the inability to provide adequate treatment to patients who were diagnosed as having pneumonia by the devices is frustrating, and improving the availability of oxygen would improve patient outcomes and access to care, and would reduce the need for referrals to other facilities.

 Fourth, our study revealed a need for increased communication and collaboration between health centres and hospitals when referrals are required. This finding is consistent with a study Southern Ethiopia where the addition of pulse oximetry to primary healthcare centres increased the number of children diagnosed with severe pneumonia but children referred to hospital often did not go due to lack of transportation.^32^ Strengthened referral networks and communication between health centres would help to ensure patient follow-up and connect patients to the care they need, consistent with other studies in Ethiopia.^36,37^ Additionally, consistent and equitably distributed resources to treat childhood pneumonia must be available at referral centres to improve access and build trust from the communities. For example, access to X-ray machines varies by facility in Ethiopia, with approximately one-third of referral and primary hospitals having access to one-half of general hospitals.^38^.

 Despite the encouraging uptake of the ChARM and RadG devices in the health system context in Ethiopia, several outstanding knowledge gaps remain on their optimal use. Discussions on the merits of using automated RR for diagnosis of pneumonia in LMICs have prevailed,^3,39-41^ but rigorous evidence is needed to understand the impact on pneumonia management and outcomes.^13^ Therefore, quantitative data on the use of automated RR counters in the pneumonia pathway, including assessment classification, treatment, referral completion, and rational antibiotic use are needed. While the availability of the devices was perceived to improve care seeking behavior and clinical management in this study, cost is a significant factor in the implementation of automated RR counters and further evidence is needed on costing within broader budgets and return on investment.^31^ This should include costs related to supplies (drugs, oxygen and device procurement) and human resources (training, follow-up) that are adaptable by differences in regional health systems.

###  Strengths and Limitations

 This study’s strengths include its diverse sample of participants, which allowed for the identification of health system bottlenecks and key considerations related to scaling-up automated RR devices in Ethiopia. The data were collected less than six months following implementation, thereby reducing the potential for recall bias. However, limitations do exist. Conclusions reached from data representing five geographic regions from participants who were purposively selected may not be generalizable to the wider population. Ethiopia has a unique model of HEWs that may compromise generalizability outside the country. Since we captured data six months after implementation, we were unable to capture long-term impact including changes in care seeking behavior or resources. Additionally, health workers provided opinions of the caregiver and patient experiences, which could lead to erroneous interpretation. Although the acceptability of the devices has already been established in research involving caregivers in this context,^13^ additional studies inviting caregivers and patients to participate is recommended. Lastly, although we attempted to minimise social desirability bias by conducting interviews in private locations and ensuring participant confidentiality, we cannot confirm that responses given were not biased.

###  Recent Developments

 Since the implementation of the ARIDA initiative, the manufacturer has discontinued ChARM production because of a lack of market viability. Despite stand-alone automated RR counters being cost-effective, validating their accuracy remains limited, especially in those 2 months old and under. Therefore, multimodal devices which have RR counters along with other capacities (eg, Rad-G Continuous, which includes measurements for hypoxemia and temperature) have gained increased interest to support child healthcare providers.^42^ They add little or no costs when compared to pulse oximeters developed for LMICs. During the COVID-19 response in India, multimodal devices like Rad-G facilitated classification, treatment and referral for childhood pneumonia in outpatient settings, allowing for timely diagnosis while limiting physical contact for health workers and patients.^43^ Prior to the pandemic, health workers in this setting checked the child with a stethoscope and touched them to check for fever. Adoption of devices like Rad-G can help to reduce physical contact to prevent the spread of pathogens by allowing healthcare providers to maintain distance. During the COVID-19 response in Ethiopia the demand for multi-modal devices like Rad-G increased along with improved access to oxygen concentrators. Building on findings from this study and the ARIDA project more widely, UNICEF used its supply catalogue to generate and catalyze demand for oxygen therapy, backed by decision support tools and programmatic support. The ARIDA project played an important role in evolving UNICEF’s approach against pneumonia and other respiratory diseases and increasing access to oxygen.

## Conclusion

 Automatic RR counters for the decentralized diagnosis of childhood pneumonia could have positive impacts on improving the quality of diagnosis and management of pneumonia in children. However, the study has shown that a health system approach is required to ensure all steps along the pneumonia pathway are adequate, including drug and oxygen supply, community engagement, health worker strengthening including training and support, and referral pathways. The introduction and scaling of innovations (such as automated RR counters) provides an opportunity for broader health system strengthening of all relevant components of the pneumonia care continuum.

## Acknowledgements

 We gratefully acknowledge the study participants who volunteered their time to share their expertise with us as well as the researchers from the Frontieri research group who conducted the interviews, transcription and translation. We also acknowledge the facilitation and time given to this study by the UNICEF Ethiopia country office, the FMoH and the respective field offices and health bureaus. This study was supported by a UNICEF grant provided by the La Caixa Foundation. We gratefully acknowledge their support. The funders played no role in the design, evaluation or interpretation of the study.

## Ethical issues

 We have no ethical issues to declare. All participants were voluntary and provided written, informed consent.

## Competing interests

 Authors declare that they have no competing interests.

## Funding

 This study was made possible through a grant to UNICEF by the La Caixa Foundation. We acknowledge their support. The funders had no influence on the study design, execution, or interpretation of the results of this study for this manuscript.

## Supplementary files

Supplementary file 1. Application of the Modified Tanahashi Bottleneck Framework.
