# Supplementary material for: Health System Considerations for Community-Based Implementation of Automated Respiratory Counters to Identify Childhood Pneumonia in 5 Regions of Ethiopia: A Qualitative Study
Source: Int J Health Policy Manag. 2023 Oct 18;12:7385. doi: 10.34172/ijhpm.2023.7385 (PMC10699823; doi:10.34172/ijhpm.2023.7385)
Supplement: Supplementary file 1 — Application of the Modified Tanahashi Bottleneck Framework. [file ijhpm-12-7385-s001.pdf]

Article title: Health System Considerations for Community-Based Implementation of Automated Respiratory Counters to Identify Childhood Pneumonia in 5 Regions of Ethiopia: A Qualitative Study

Journal name: International Journal of Health Policy and Management (IJHPM)

Authors' information: Angeli Rawat[1,2]*, Agazi Ameha[3], Jonas Karlström[1,4], Lisanu Taddesse[5], Elias Legesse Negeri[6], Anne Detjen[7], Kristoffer Gandrup-Marino[8], Noah Mataruse[8], Karin Källander[9,10], Abraham Tariku[5]

[1]UNICEF Supply Division Innovation Unit, Copenhagen, Denmark.

[2]School of Population and Public Health, University of British Columbia, Vancouver, BC, Canada

[3]UNICEF Ethiopia Country Office, Addis Ababa, Ethiopia.

[4]Global Programmes and Research, SingHealth Duke-NUS Global Health Institute, Duke-NUS, Singapore, Singapore.

[5]Federal Ministry of Health of Ethiopia, Addis Ababa, Ethiopia.

[6]Frontieri Consult, Addis Ababa, Ethiopia.

[7]Child and Community Health Unit, Health Programme Group, UNICEF, New York City, NY, USA.

[8]UNICEF Supply Division, Copenhagen, Denmark.

[9]Digital Health and Health Information Systems Unit, Health Programme Group, UNICEF, New York City, NY, USA.

[10]Department of Global Public Health, Karolinska Institutet, Stockholm, Sweden.

***Correspondence to:** Angeli Rawat; Email: angeli@alumni.ubc.ca

Citation: Rawat A, Ameha A, Karlström J, et al. Health system considerations for communitybased implementation of automated respiratory counters to identify childhood pneumonia in 5 regions of Ethiopia: a qualitative study. Int J Health Policy Manag. 2023;12:7385. doi:10.34172/ijhpm.2023.7385

Supplementary file 1. Application of the Modified Tanahashi Bottleneck Framework

1 – Application of the modified Tanahashi Bottleneck Framework.

This file describes the modification made to the Tanahashi Bottleneck Analysis Framework and the application of second level codes to the framework.

The Tanahashi model focuses on the interaction between service provision and the end users of the services. We modified the framework for the qualitative data with an emphasis on ascertaining health system successes and challenges related to implementation of the ARIDA project regionally and nationally, within the following domains: 1) Supply; 2) Demand; 3) Enabling environment;

and 4) Quality. The ‘supply’ domain included themes related to the availability of commodities and supplies (e.g., drug supply, ARIDA devices, and oxygen gas) and community engagement. The ‘demand’ theme included initial and continuous utilization (e.g., health seeking behaviour, referral pathways, scale-up and health system integration) and cultural practices/beliefs (e.g., caregiver acceptability of the intervention). The ‘enabling environment’ theme encompassed management and coordination (e.g., supportive supervision and training), budget and expenditure (e.g., financial resources needed to absorb the intervention into the health system), legislation and policy (e.g., evidence and actors needed for policy engagement and IMNCI integration). Lastly, the domain ‘quality’ included the effective coverage of quality services (e.g., staff satisfaction and motivation, device malfunction).

Table S1: Application of the adapted Tanahashi bottleneck analysis framework to thematic content from Key Informant Interviews

Domain	*Application*	*Tracer(s)*	*Code Groups*
**Supply**	Availability of commodities/ essential materials/inputs	Amoxicillin availability<br><br>Gentamicin availability<br><br>Oxygen supply<br><br>ChARM	*Supply_Availability of Commodities/EM-Amoxicillin*<br>• S_Amox-Availability<br>• S_Amox-Stockout/challenges<br>*Supply_Availability of Comm/EM-Gentamicin*<br>• S_Gent-Availability<br>• S_Gent-Stockout/challenges<br>*Supply_Availability of Comm/EM-Oxygen*<br>• S_O2-Availability<br>*Supply_Availability of Comm/EM-ChARM*<br>• S_ChARM-Success<br>• S_ChARM-Challenge<br>• S_ChARM-Charge duration<br>• S_ChARM-Numbers of devices available

			• S_ChARM-Speed<br>• S_ChARM-Accuracy<br>• S-ChARM-Recommendation
		Rad-G	*Supply_Availability of Comm/EM-Rad-G*<br>• S_Rad-G-Success<br>• S_Rad-G-Challenge<br>• S_Rad-G-Charge duration<br>• S_Rad-G-Numbers of devices available<br>• S_Rad-G-Speed<br>• S_Rad-G-Accuracy<br>• S-Rad-G-Recommendation
		Stockout general (non-specified drug)	Supply_Availability of Comm/EM-stockout/barriers general
	Availability of community outreach activities	Community engagement success and challenges	*Supply_Availability of Comm Outreach-Comm Engagement*<br>• S_CommEng-Success<br>• S_CommEng-Challenge<br>• S_CommEng-How<br>• S_CommEng-Recommendation
**Demand**	Initial utilization	Health seeking behaviour/	Demand_Initial Utilization-Community/Population Uptake

		population uptake of intervention	• D_comm/popuptake-success<br>• D_comm/popuptake-challenge<br>• D_comm/popuptake-recommendations<br>• D_comm/popuptake-health seeking
	Continuous utilization/ Financial access (direct or indirect costs)	Referral pathways (cost: time or finances to continuous utilization)	Demand_Continuous Utilization/Financial_Referral Pathways<br>• D_Referral pathway-success<br>• D_Referral pathway- challenge<br>• D_Referral pathway-time/distance<br>• D_Referral pathway-recommendation<br>Demand_Continuous Utilization/Financial_Referral Oxygen<br>• D_O2_Referral time/distance<br>• D_O2_Referral success<br>• D_O2_Referral challenges
	Continuous utilization	Scale-up and health system integration	Demand_Continuous Utilization-Scale Up/HS integration
	Cultural practices/beliefs	Caregiver responses and child reactions to ARIDA devices (acceptability)	Demand_Cultural Practices/Beliefs-Caregiver reaction<br>• D_caregiver-positive<br>• D_caregiver-negative<br>• D_caregiver-mixed<br>• D_caregiver-response if negative<br>Demand_Cultural Practices/Beliefs-Child reaction & solutions ARIDA<br>• D_childreaction-positive<br>• D_childreaction-mixed

			• D_childreaction-negative<br>• D_childreaction-response if negative; impact or solution
**Enabling environment**	Management/ Coordination	Supportive supervision<br><br>Training	Enabling Environment_Management/Coordination-Supportive Supervision<br>• EE_SS-Success<br>• EE_SS-Challenge<br>• EE_SS-Recommendations<br>Enabling Environment_Management/Coordination-Training<br>• EE_Training_Success<br>• EE_Training_Challenge<br>• EE_Training_Recommendations<br>• EE_ToT
	Budget/ Expenditure	Financial resources to absorb into HS nationally	Enabling Environment_Budget/Expenditures-Finances
	Legislation and policy	Policy engagement (who and how), evidence needed	Enabling Environment_Legislation/Policy-Policy Engagement-evidence & support needed<br><br>Enabling Environment_Legislation/Policy-Policy Engagement-how<br><br>Enabling Environment_Legislation/Policy-Policy Engagement-rec/integration<br><br>Enabling Environment_Legislation/Policy-Policy Engagement-who

	Legislation and policy	IMNCI integration	Enabling Environment_Legislation and Policy-IMNCI integration
**Quality**	Effective coverage of quality services	Staff satisfaction and motivation	Quality_Effective Coverage_Staff Satisfaction
		ChARM malfunction	Quality_Effective coverage quality services-ChARM malfunction
		Rad-G malfunction	Quality_Effective coverage quality services-Rad-G malfunction
